# Editorial: AI taking actions in the physical world - Strategies for establishing trust and reliability

**DOI:** 10.3389/fnbot.2023.1200802

**Published:** 2023-04-28

**Authors:** Sirko Straube, Ryuji Yamazaki-Skov, Raul Hakli

**Affiliations:** ^1^Robotics Innovation Center, Deutsches Forschungszentrum für Künstliche Intelligenz (DFKI), Bremen, Germany; ^2^Institute for Open and Transdisciplinary Research Initiatives, Osaka University, Toyonaka, Japan; ^3^Department of Practical Philosophy, University of Helsinki, Helsinki, Finland

**Keywords:** Artificial Intelligence, Robotics, trustworthy AI, reliability, human machine collaboration, hybrid teams

We are living at a time, when Artificial Intelligence (AI) and Robotics are changing our everyday and working life more and more. Sometimes this passes rather unnoticed, e.g., when streaming providers use AI algorithms to propose movies and series that fit to our user profile, sometimes it is obvious, e.g., when we try to enable autonomous driving in our streets. Especially when AI decisions are clearly affecting our living and working environments, it is of vital importance that human users experience the sources of these decisions as trustworthy and reliable. The question of how to enable this is exactly at the heart of this Research Topic as it is also part of a huge and very diverse landscape of research that is currently ongoing. Taking this heterogeneity of approaches into account, we divided the topic to be addressed into the fields of (i) robots or simply actuators acting in a real-world environment, (ii) AI methods to establish knowledge representations and experience, (iii) the essential question of how to handle uncertainty in models and reality in decision making, (iv) the issue of having social AI capabilities for interaction with humans, and finally (v) how ethical and philosophical perspectives can be addressed appropriately, since these technological advances will change our interactions within our societies, be it in the contexts of work or everyday life.

Since making AI reliable and establishing trust is so important in all kinds of possible applications, we therefore addressed a wide field of research activities. This kind of broadness is in retrospect both the strength and the weakness of this Research Topic. We see from the results that the intended theme indeed shows that we are looking from very different perspectives on the same issue and it illustrates the heterogeneity of the approaches that are currently under research. On the other hand, we need more integrative and unified AI approaches that are also interoperable, so that one application can interface another one. A high level of reliability of and introspection on AI would be a fundamental building block for such a unification of AI approaches, since communication of uncertainties or even reasoning about failures would support interfacing with AI systems and, most importantly, strengthen the trust a human user would establish in the application (in contrast to a black box functionality). However, such a unified AI concept or strategy is still not common sense, and we can also not derive the overall tendencies from the current issue.

Still, by looking at the contributions in the Research Topic from a higher perspective, some principles of current research directions are seen very nicely. Reliability is always depending on knowledge when an AI (or a robot) encounters unknown situations. When the experience in the real world is missing, internal simulations can be applied to fill the gap and to establish at least basic strategies of the AI to react in new situations. A study investigating exactly this gap between physical world and simulation is provided here by Tiedemann et al.. Additionally, simulations are the first step for an internal model of the AI itself, which would be a prerequisite for being transparent about decision making. Geraci et al. take up this strategy by discussing inner speech of robotic systems as a mechanism to improve human trust by increasing the transparency of decisions made. If experience is missing at all, then an idea often pursued is to transfer existing knowledge (or learned parameters in an algorithm) from a known application to an unknown situation. Such transfer learning is used in many applications. In this Research Topic we have a very practical example coming from biomedical question answering provided by Zhu et al.. Finally, AI algorithms acting in the real world cannot only react to incoming data, instead they have to predict what will happen—just as the human brain has to make predictions about upcoming behaviors of other humans and—in the future also—robots interacting with them. This key issue is tackled by the study about movement prediction by Veselic et al.. All of these studies are addressing current research directions to sustainably influence how we will interact and interface with AI systems in the future.

The changes that are addressed in this Research Topic are just about to happen and accordingly the strategies to implement AI technologies to assist humans in their everyday life are still being developed with underlying high dynamics. The latest advances that we see in very deep neural networks and large language models trained on massive data make it very obvious that the technological possibilities increase much faster than our ideas on how to actually use the technology in a responsible and beneficial way. We see that it is therefore now more important than ever to establish mechanisms that enable users to interact with an AI in a reliable and trustful way.

It was an exciting journey to establish this Research Topic.

## Author contributions

All authors listed have made a substantial, direct, and intellectual contribution to the work and approved it for publication.

